# Supplementary material for: A deep residual model for characterization of 5D spatiotemporal network dynamics reveals widespread spatiodynamic changes in schizophrenia
Source: Front Neuroimaging. 2023 Feb 1;2:1097523. doi: 10.3389/fnimg.2023.1097523 (PMC10406273; doi:10.3389/fnimg.2023.1097523)
Supplement: Supplementary file 1 [file Data_Sheet_1.docx]

Supplementary Material

# Spatial variation comparison over groups for all 53 networks

In order to study group differences between healthy-control and schizophrenia subjects considering all 53 brain networks, we drew spatial maps averaged over time and subject for each group along with voxel-wise t-Test and also masked-out irrelevant regions in order to focus on ROI. Moreover, we inspected spatial deviation over time, averaged over subjects to see spatial variability differences between groups as is shown in figure 1 to 9.

Supplementary Figure 1. The figure shows spatial maps together with spatial deviation maps for both groups of health-control and schizophrenia along with subtraction and 2 sample T-test maps for cerebellar and auditory networks.

Supplementary Figure 2. The figure shows spatial maps together with spatial deviation maps for both groups of health-control and schizophrenia along with subtraction and 2 sample T-test maps for sensori-motor networks.

Supplementary Figure 3. The figure shows spatial maps together with spatial deviation maps for both groups of health-control and schizophrenia along with subtraction and 2 sample T-test maps for sensori-motor networks.

Supplementary Figure 4. The figure shows spatial maps together with spatial deviation maps for both groups of health-control and schizophrenia along with subtraction and 2 sample T-test maps for visual networks.

Supplementary Figure 5. The figure shows spatial maps together with spatial deviation maps for both groups of health-control and schizophrenia along with subtraction and 2 sample T-test maps for default mode networks.

Supplementary Figure 6. The figure shows spatial maps together with spatial deviation maps for both groups of health-control and schizophrenia along with subtraction and 2 sample T-test maps for cognitive control networks.

Supplementary Figure 7. The figure shows spatial maps together with spatial deviation maps for both groups of health-control and schizophrenia along with subtraction and 2 sample T-test maps for cognitive control networks.

Supplementary Figure 8. The figure shows spatial maps together with spatial deviation maps for both groups of health-control and schizophrenia along with subtraction and 2 sample T-test maps for cognitive control networks.

Supplementary Figure 9. The figure shows spatial maps together with spatial deviation maps for both groups of health-control and schizophrenia along with subtraction and 2 sample T-test maps for subcortical networks.
