# Supplementary figures and images for: A deep residual model for characterization of 5D spatiotemporal network dynamics reveals widespread spatiodynamic changes in schizophrenia
Source: Front Neuroimaging. 2023 Feb 1;2:1097523. doi: 10.3389/fnimg.2023.1097523 (PMC10406273; doi:10.3389/fnimg.2023.1097523)

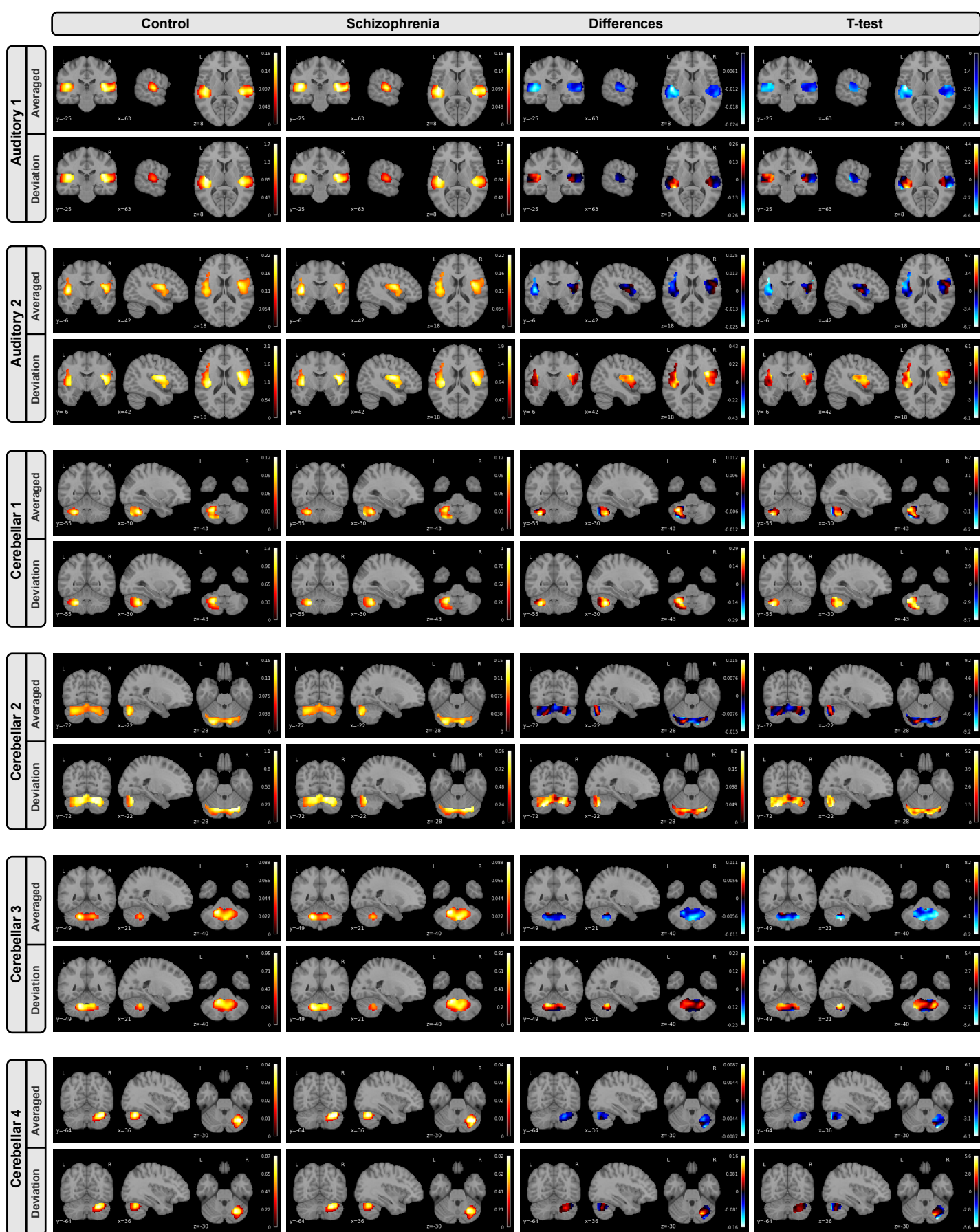

Supplement: Supplementary file 2 [file Data_Sheet_1.PDF]

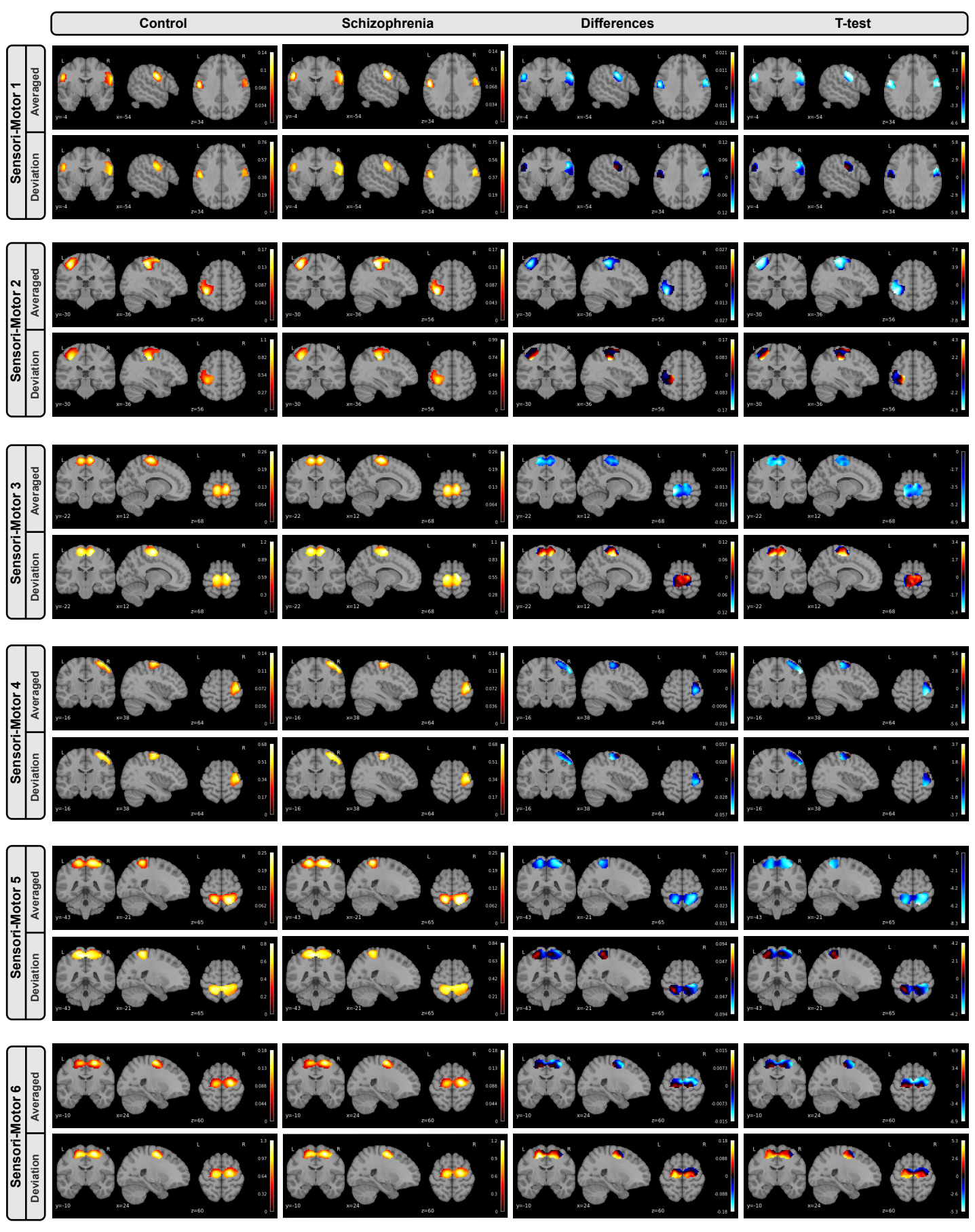

Supplement: Supplementary file 3 [file Data_Sheet_2.PDF]

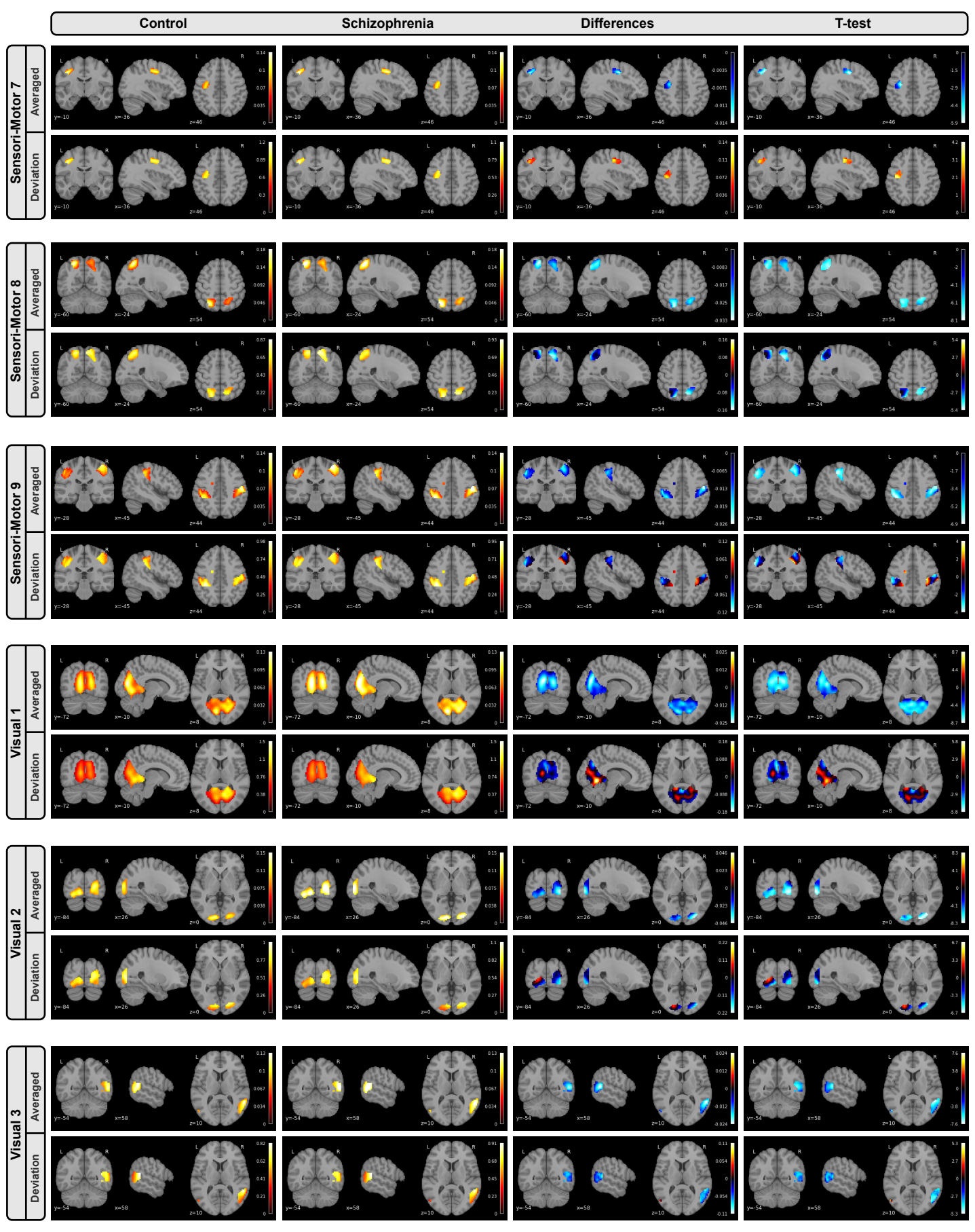

Supplement: Supplementary file 4 [file Data_Sheet_3.PDF]

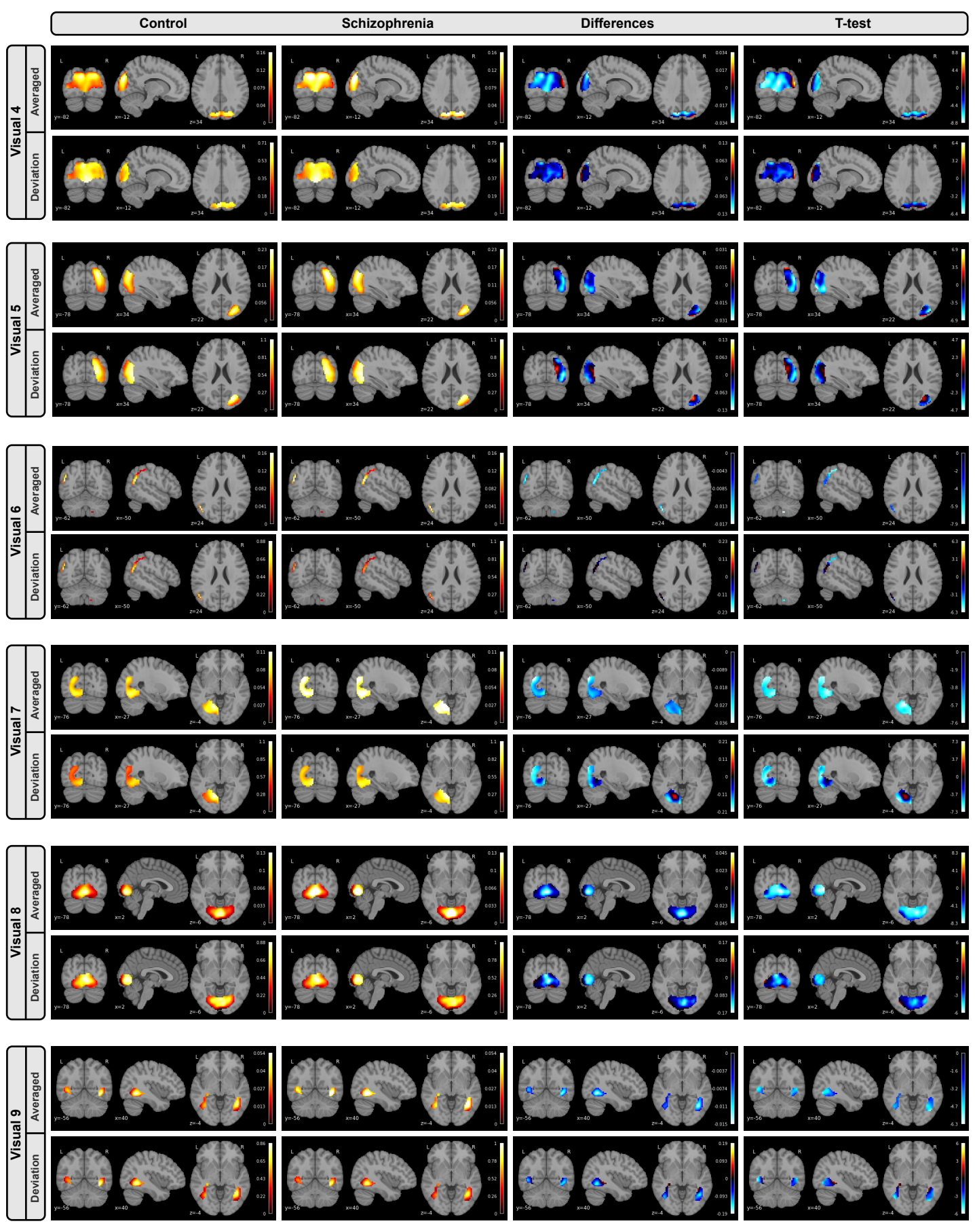

Supplement: Supplementary file 5 [file Data_Sheet_4.PDF]

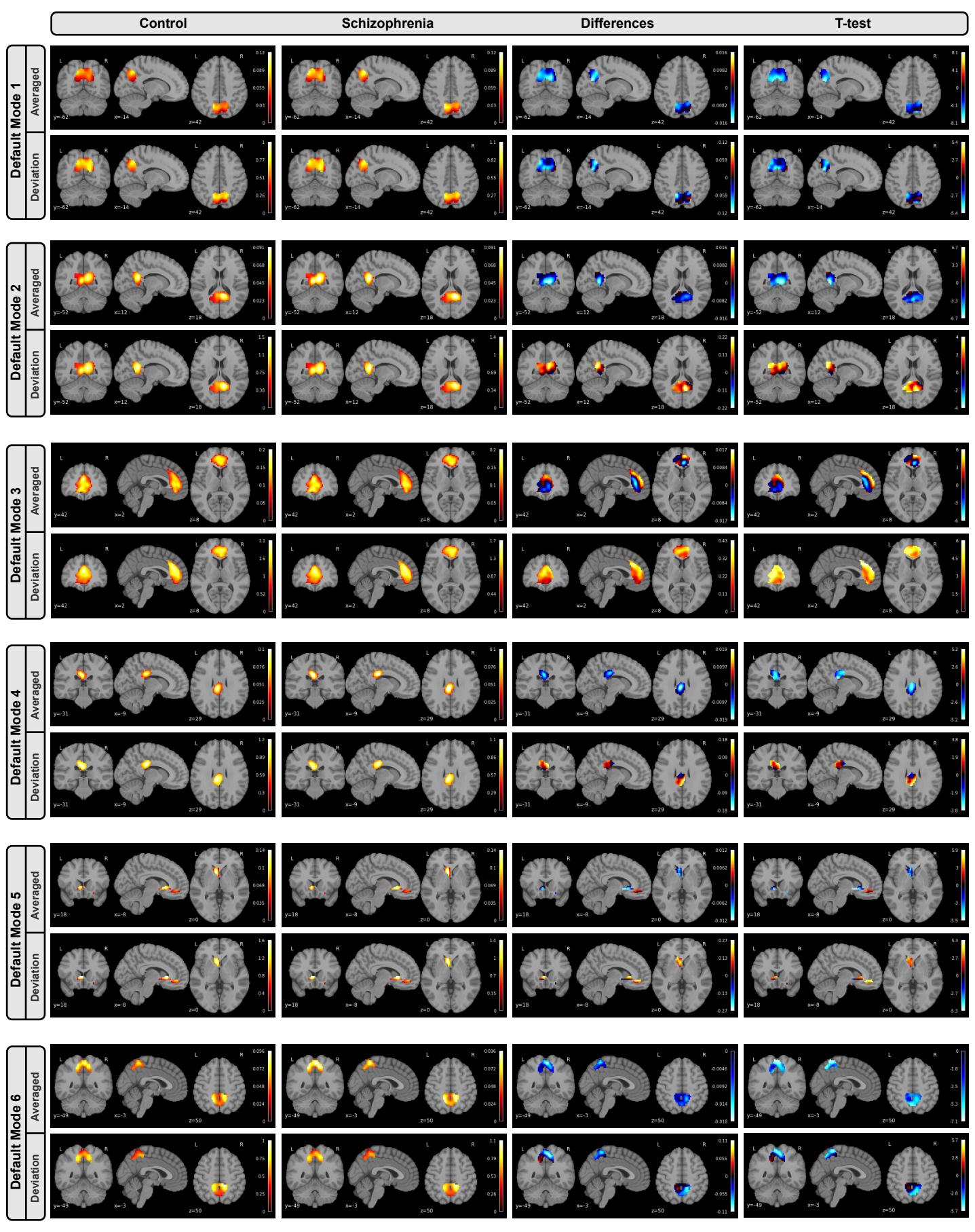

Supplement: Supplementary file 6 [file Data_Sheet_5.PDF]

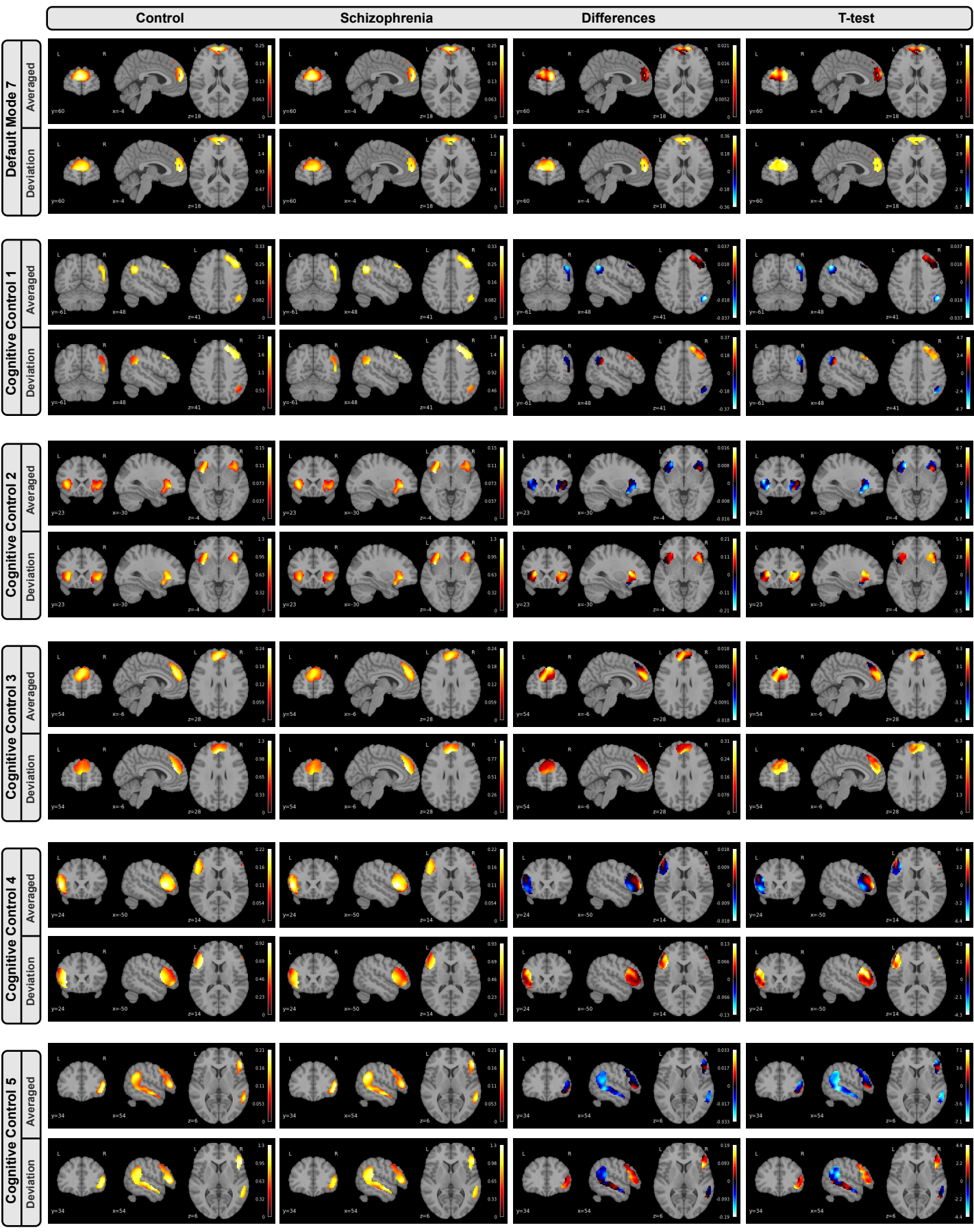

Supplement: Supplementary file 7 [file Data_Sheet_6.PDF]

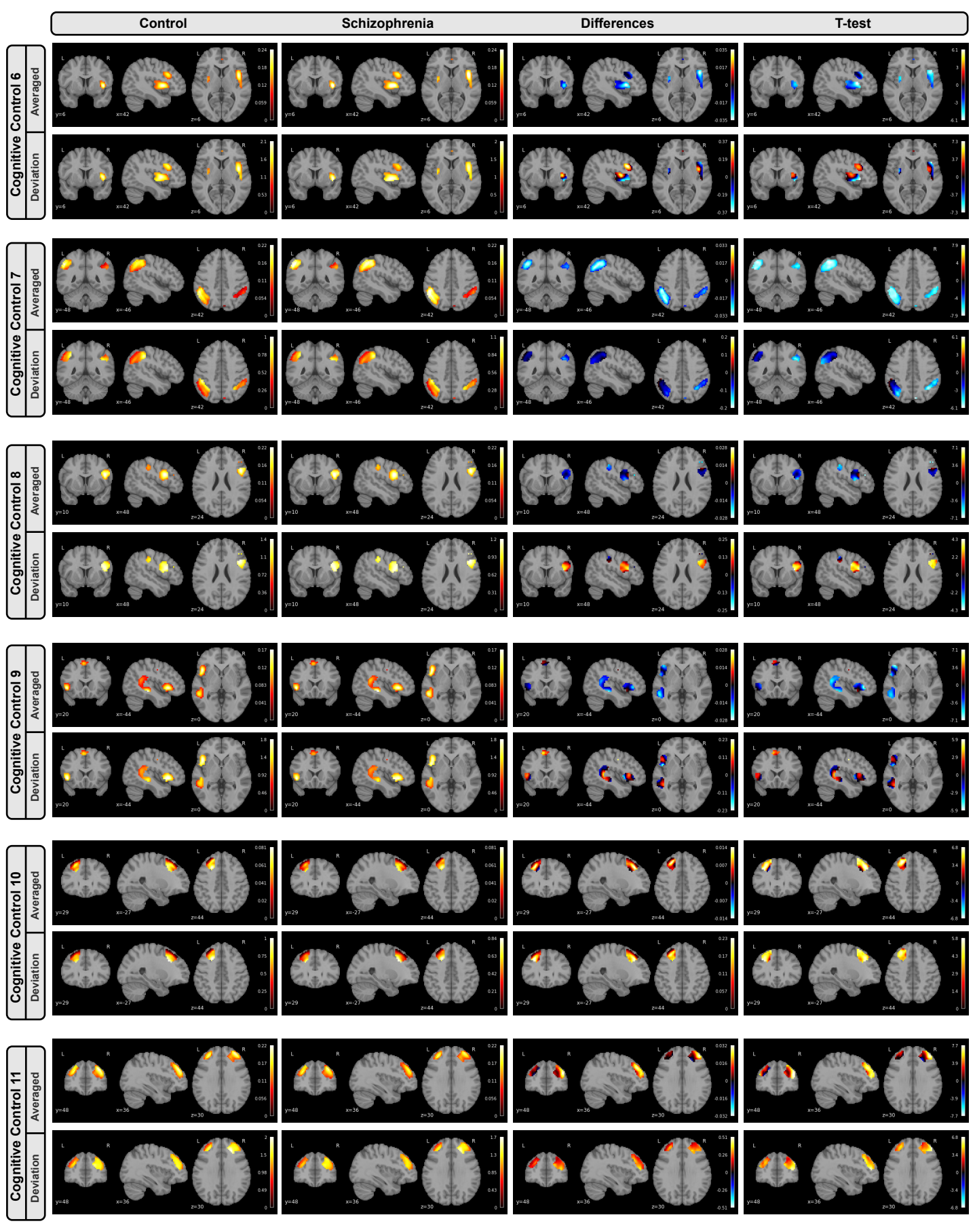

Supplement: Supplementary file 8 [file Data_Sheet_7.PDF]

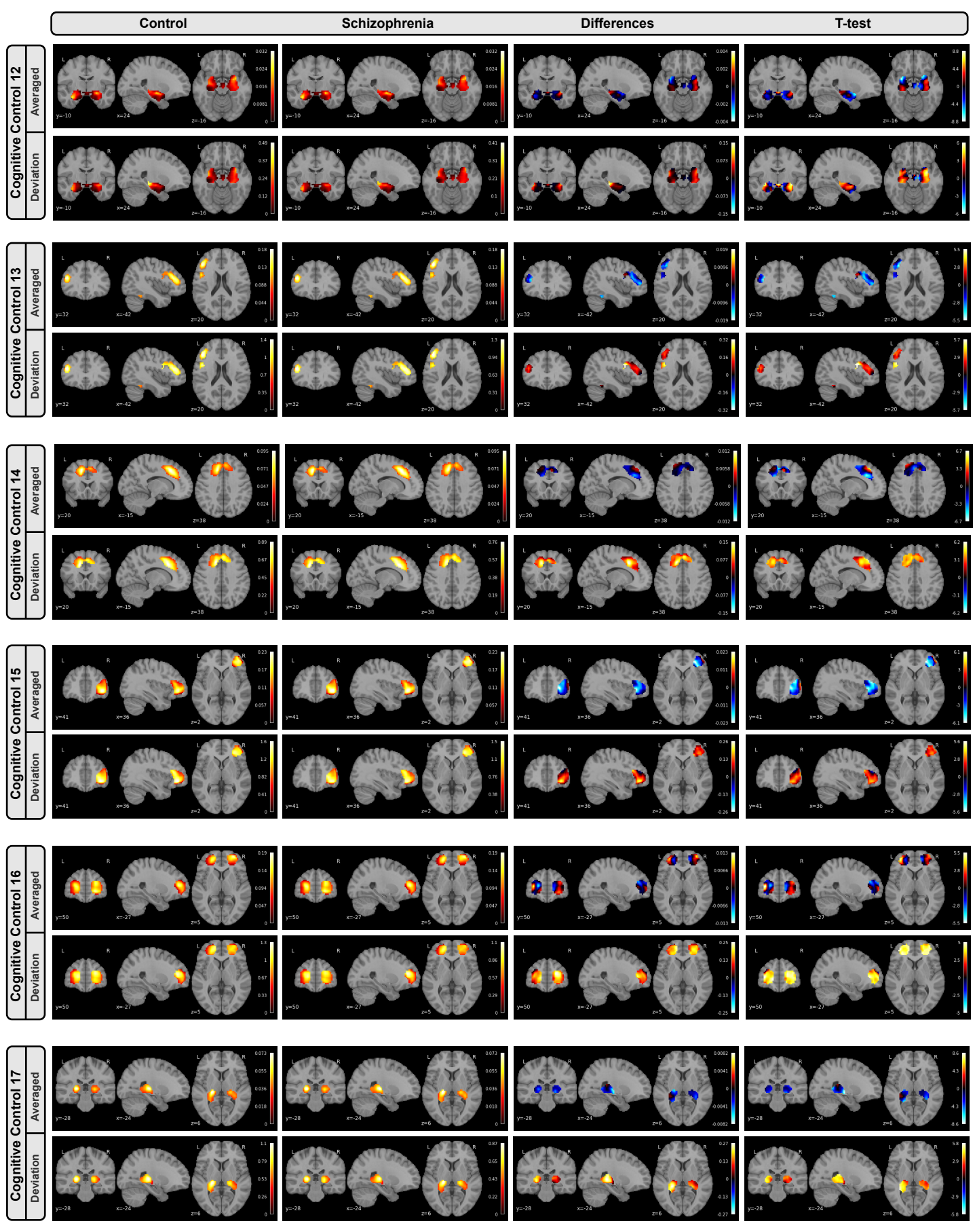

Supplement: Supplementary file 9 [file Data_Sheet_8.PDF]

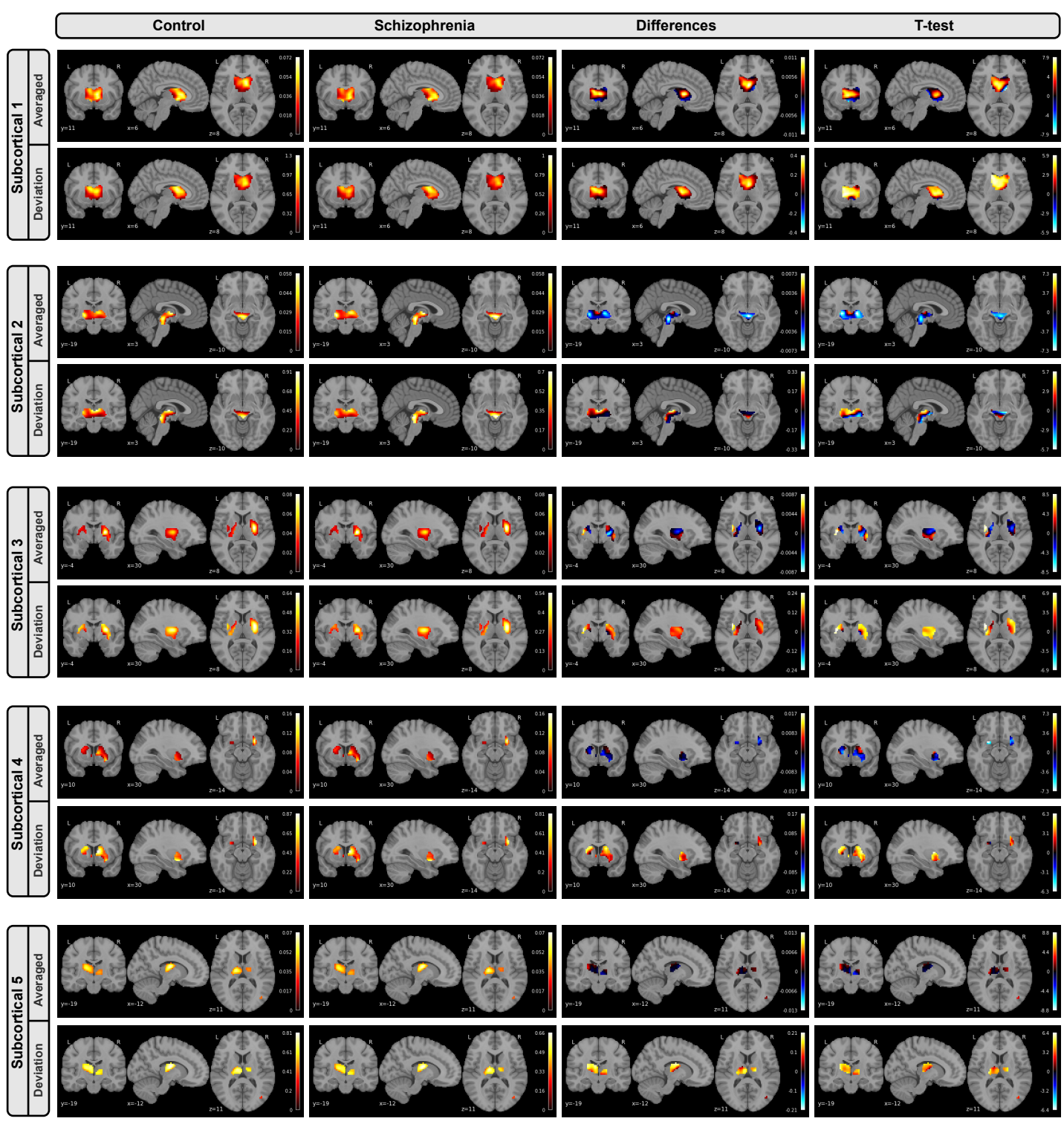

Supplement: Supplementary file 10 [file Data_Sheet_9.PDF]
